# How can green credit decrease social health costs? The mediating effect of the environment

**DOI:** 10.3389/fpubh.2023.1121154

**Published:** 2023-01-20

**Authors:** Yanbo Rong, Jinyan Hu

**Affiliations:** School of Economics, Qingdao University, Qingdao, Shandong, China

**Keywords:** green credit, social health costs, environmental pollution, quantile regression (QR), mediating role

## Abstract

Green credit plays an important role in environmental protection and residents' health. This paper discusses the impact path of green credit on social health costs with the help of a quantile regression. The implementation of a green credit policy can decrease social health costs in China, and green credit works best in the economically developed Eastern region. As the quantile increases, so gradually does the absolute value of the green credit coefficient. This result proves that for provinces with rich per capita financial health expenditures, green credit plays a greater role in decreasing social costs, a conclusion also supported by our robustness test. In addition, we find that environmental pollution plays a mediating role in the path of green credit affecting health, and this finding is verified in the green credit and health general equilibrium model. Based on these findings, the government should encourage the active innovation of green credit products, and the banking industry should develop personalized green credit products for specific pollutant types or industries while decreasing government pressure.

## 1. Introduction

Underlying rapid economic development is the massive cost of environmental resources and medical and health care. According to the World Bank, although energy consumption per unit of GDP has decreased, medical expenditures have increased rapidly. At present, pollution is the world's largest environmental risk factor for disease and premature death, especially in low- and middle-income countries. In 2019, 9 million people died from pollution, equivalent to 1/6 of the global death toll^1^. Pollution and pollution-related consequences are the main inducements that have a massive impact on health (1), having a negative effect on the mental health of the public, and decreasing subjective wellbeing and mental health. Environmental pollution is closely related to the allocation of credit resources. Green credit supports low energy consumption, low emissions, low pollution, and high efficiency business behaviors, the last of which is an important practice of guiding green development through the rational allocation of credit funds (2). Improving the allocation of credit capital to green industries can curb the impact of environmental pollution on residents' health, which is conducive to achieving win–win economic and environmental goals (3, 4).

China's environmental pollution has always been very serious. Outdoor air pollution has become the fourth leading lethal risk factor (5), and the number of deaths due to environmental pollution in China ranks second globally (6). As China has industrialized, infrastructure construction and the heavy chemical industry have consumed large amounts of mineral resources and fossil fuels and have had an important impact on people's health. Thus, the health of Chinese households is directly related to the rapid development of resource-intensive industries as a result of traditional credit (7). China proposed supply-side structural reform in 2015, hoping to optimize the allocation of financial resources and improve residents' health. The 13th 5-Year Plan clearly included structural adjustment and environmental protection as the overall goal of macro control. The 19th National Congress of the CPC made it clear that “we should treat the ecological environment like life” and listed environmental protection as one of the “three tough battles.” Under the current multiple difficulties of prominent financial and credit mismatch, worsening ecological environment and increasing household health expenditures, how to improve the quality of economic growth and achieve sustainable growth are core issues for China's future economic development.

This paper makes three main contributions. Firstly, we consider the new path of environmental pollution to explain the impact of green credit on social health costs. The literature has generally been based on the impact of green credit on enterprises' green technology innovation to improve environmental quality. Alternatively, some research has been done on the harm environmental pollution does to residents' health. The promotion of green credit in financial fields can decrease financial pressure and release further funds for health. More importantly, residents' health can be improved by improving environmental quality. This fact also conforms to green credit and health general equilibrium theory, which verifies the intermediary role played by the environment. Secondly, this paper uses a quantile regression (QR) to test the impact of green credit on social health costs under different quantiles. The independent variable has a specific regression curve on each characteristic quantile of the dependent variable, which is the advantage of this model. For provinces with higher per capita financial health expenditures, green credit plays a greater role in decreasing social costs. Finally, our study fully considers heterogeneity. The impact of green credit on social health costs shows an obvious geographical ladder distribution, with the largest effect in the Eastern region, followed by the Central region, and an as-yet-undiscovered effect in the Western region. This conclusion is helpful for banks as they issue targeted green credit policies and decreases social health costs in underdeveloped areas.

The rest of this study is organized as follows. Section 2 covers the literature review. Section 3 outlines the theoretical model. Section 4 describes the methodology. Section 5 describes the data. Section 6 presents a discussion of the empirical findings. Section 7 summarizes the results and discusses some policy implications.

## 2. Literature review

Green credit can affect national health spending in two ways. On the one hand, green credit can optimize the industrial structure and cause more people to engage in tertiary industries, thus directly improving residents' health levels. On the other hand, green credit can improve environmental conditions and play a positive role in public health. Therefore, considering the intermediary effect of the environment, this paper expands the existing literature in three ways: green credit and health, green credit and the environment, the environment and health.

### 2.1. Green credit and health

As an important guarantee for the real economy, sustainable development, and a country's core competitiveness, credit business can play an indispensable role in building a community of human health. Frederik et al. (8) found that the debt burden is steadily increasing of low- and middle-income countries, coupled with the economic recession of COVID-19, which combined might nullify the necessary asset health expenditures. Tuohy et al. (9) showed that the impact of private financing on publicly funded health care systems depends on the construction of the relationship between public and private financing. The authors think that the increase in private health expenditures partly replaces public finance. Leatherman (10) showed that microfinance institutions have advantages in developing health financing programs, which can expand poor people's existing choices, and prevent the risk of poverty caused by disease. Maurya and Asher (11) revealed that India spent 3.7% of its GDP on health care, but the health care results were not commensurate with the expenditures. Therefore, increasing public health expenditures alone does not improve health outcomes unless the inefficiency of existing public and private health financing arrangements is addressed.

The research in China has drawn a relatively consistent conclusion: green credit has a positive impact on health. Liu and Guo (12) discussed the significant positive effect of inclusive finance on public health based on China's data. Hu et al. (13) found that China's green credit has significant effects on the transformation of the industrial structure. Green credit mainly influences population health through the industrial structure. Zhu (14) combined green credit, technological innovation, industrial structure upgrades, and population health; theoretically analyzed the impact of green credit and technological innovation on industrial structure upgrades and population health; and analyzed how green credit affects population health through technological innovation and industrial structure upgrades.

### 2.2. Green credit and environmental conditions

Green credit has effectively decreased the pollution and energy consumption of high-emission enterprises (15), and the literature has generally concluded that green credit can greatly improve environmental quality. Based on data from the BRICS countries (i.e., Brazil, Russia, India, China, and South Africa), Wang et al. (16) revealed that inclusive finance is a catalyst to promoting the growth of renewable energy investment and decreasing carbon emissions. Zeng et al. (17) found that green finance, EU consumption, and technological innovation performed well in protecting the environment by decreasing carbon emissions. Muhammad et al. (18) used data from South Asia to show that green bonds, decreasing greenhouse gas emissions, and green economic development played an important role in green financial development. Zhang et al. (19) conducted a dynamic relationship study on samples from 49 countries and showed that green finance could effectively mitigate environmental pollution and climate change. Accelerating the development of green finance is the primary driving force for achieving sustainable development.

Researchers have also reached a consensus on green finance in China. Qiao et al. (20) found that China's financial development is positively related to environmental pollution, and further noted that China is in the first stage of an “environmental dividend.” To enter the second stage of “sustainable finance,” China should increase the reasonable income of “green finance” and establish a unified national carbon trading market. Sun et al. (21) found that China's green credit policy has greatly encouraged enterprises, especially those that rely heavily on external financing, to decrease water pollution. Huang et al. (22) showed a significant positive auto-correlation between green finance and green innovation. Tang et al. (23) discussed the relationship between green finance and the ecological environmental quality of the Yangtze River Economic Belt and found a significant positive impact. Xu and Zhu (24) proved that China's overall green governance index and green financial policies have significantly decreased environmental pollution. In addition, some literature has studied the ways green finance restricts polluting enterprises, which has increased green technology innovations (7, 25). Chen et al. (26) affirmed that financial development can ease the financing constraints faced by innovative activities and promote green technological innovation. Hong et al. (27) considered that green credit guidance mainly restricts green technology innovation by decreasing debt financing rather than through financing constraints. Yang and Zhang (28) thought that implementing green credit policies significantly inhibited the long-term financing of heavily polluting enterprises but allowed heavily polluting enterprises to expand their short-term financing. Chai et al. (29) also used data from Chinese enterprises to support this view.

However, green finance does not invariably improve the environment: at different economic development levels, the influence differs (30). For example, Zhong (31) realized that digital finance's environmental improvement has a threshold, after which an acceleration effect can result.

### 2.3. Environment and health

Pollution forces humans to face massive health costs and survival threats, leading to the rapid depreciation of health human capital. Thus, pollution constitutes an important source of inequality in economic and social development. The health demand function first proposed by Grossman (32) focused on environmental pollution and residents' health from an economic perspective. Based on this function, Cropper (33) and Wagstaff (34) introduced environmental pollution into the model as an important variable that significantly affects human health. Since then, many studies have affirmed the importance of environmental quality and the role of public health functions in improving public health in cooperation with local governments. Scally and Perkins (35), Welsch (36), and Knibbs et al., (37) found that long-term exposure to high particle matter concentrations increased the risk of children suffering from asthma. Awais and Tariq (38) proved that the building environment was linked to human health through physical activity opportunities, and pollutants such as PM10 also increased risks (39–41). Some literature has discussed the harm environmental pollution poses to residents' physical and psychological wellbeing. Albertini et al. (42) showed that environmental pollution is the major cause, other than age, of the increase in the health depreciation rate and the accelerated decrease in the health stock. Ivanova (43) considered that clean air is a prerequisite for human health and happiness.

The research on China has also drawn a relatively consistent conclusion. Zhao et al. (44) thought that China's massive energy consumption and low energy efficiency have caused severe environmental pollution and posed a great threat to the national health level. The general occurrence of health complications caused by cumulative environmental pollution in China is on the rise. Wang et al. (45) found that external environmental pollution and subjectively perceived pollution are negatively correlated with public health. Specifically, air pollution and domestic waste pollution have significant associations, mainly with public mental health. Wang et al. (46) proved that the environmental health indicators of atmospheric pollution in Central and Eastern China are low, indicating a serious environmental health condition. Chen et al. (47) revealed that the differential heating policy between the north and the south of China was very likely to cause air pollution in the north, which would led to a decrease in residents' average life span in this region by approximately 5.5 years.

Regarding the research on green credit, the existing literature mostly discusses its impact on the green innovation or transformation and upgrading of polluting enterprises, which are activities that improve environmental quality. However, few studies have examined the impact of green credit on health. More studies have been focused on the substitution of financial health expenditures from the private financing perspective, and little research has been conducted from an empirical perspective on the direct and indirect pathways. This paper not only considers the direct impact of green credit on social health costs but it also discusses the mediating effect of environmental pollution, which enriches the existing research.

## 3. Green credit and health general equilibrium model

According to the family production function (32), a simplified health production function can be expressed as:


(1) $$\begin{array}{l} H=G\left(X\right)=G\left(X_{1},X_{2},\cdots \;,X_{n}\right) \end{array}$$

where *X*_*i*_ is the factors influencing health, including environment (E), health expenditures (M0), population structure (P), and other variables (Z). Then, the health production function can be expressed as:


(2) $$\begin{array}{l} H=G\left(E,M,P\right)=\Omega \prod \overset{\alpha_{i}}{\underset{i}{E}}\prod \overset{\beta_{i}}{\underset{i}{M}}\prod \overset{\gamma_{i}}{\underset{i}{P}}\prod \overset{\lambda_{i}}{\underset{i}{Z}} \end{array}$$

where *α*, *β*, *γ*, *and λ* are the corresponding elasticity coefficients, and Ω is the estimated value of initial social health. Taking a logarithm of Equation (2), we obtain:


(3) $$\begin{array}{lll} LnH & = & Ln\Omega +\sum \alpha_{i}\left(LnE_{i}\right)+\sum \beta_{i}\left(LnM_{i}\right)+\sum \gamma_{i}\left(LnP_{i}\right) \end{array}$$

$$\begin{array}{ll} + & \sum \lambda_{i}\left(LnZ_{i}\right) \end{array}$$

We expand the financial sector based on neoclassical growth theory with resource constraints. Suppose an economy has two enterprise types that provide intermediate products. *h* represents polluting enterprises, which are characterized by heavy assets, such as steel and petrochemical; *l* represents clean enterprises, which are characterized by light assets and high technological levels, such as electronic components. The production functions of the two enterprise types are:


(4) $$\begin{array}{l} Y_{h}=N^{\beta_{1}}K_{h}^{\beta_{2}};Y_{l}=A\left(t\right)K_{l}^{\gamma } \end{array}$$

where *h* enterprises consume natural resources *N* and material capital *K*_*h*_, *l* enterprises do not consume natural resources (actually, they consume fewer, a fact that is simplified here), and use only healthy human capital H and material capital *K*_*l*_, with technological progress parameter A. In the consideration of technological progress, A is a function of time *t*. Production is also affected by the environment. Following the assumption of Bowenberg and Simus (48), P represents pollution, and as it deepens, the output decreases more. Thus, the production function is as follows:


(5) $$\begin{array}{l} Y=Y_{h}^{\alpha_{1}}Y_{l}^{\alpha_{2}}P^{-\alpha_{3}},\alpha_{1},\alpha_{2},\alpha_{3}>0 \end{array}$$

Under steady economic conditions, we obtain^2^:


(6) $$\begin{array}{l} g_{E}=\frac{\left(\sigma -1\right)}{\left(1+\omega \right)}\frac{1}{\sigma }\left[\mu_{2}\left(\frac{Y}{K}\right)-\rho \right] \end{array}$$

where gE indicates variable change, and if it increases in the positive direction, the environment improves. Green credit ξ acts on the material capital of environmental protection enterprises, where a mean *K*_*l*_ = ξ*K*. Substituting production Equation (5) considering green credit into Equation (6), while taking the logarithms, we obtain Equations (7) and (8):


(7) $$\begin{array}{lll} g_{E} & = & \frac{\left(\sigma -1\right)}{\left(1+\omega \right)}\frac{1}{\sigma }\left[\mu_{2}h^{\alpha_{3}}A^{\alpha_{2}}N^{\mu_{1}}K_{h}^{\mu_{2}-1-\alpha_{2}\gamma }K_{l}^{\alpha_{2}\gamma }\left(1-\xi \right)-\rho \right] \end{array}$$

(8) $$\begin{array}{l} \ln g_{E}\;=\;\phi +\alpha_{3}\ln h+\left(\alpha_{1}\beta_{1}-\alpha_{3}\right)\ln N+\left(\alpha_{1}\beta_{2}-1\right)\ln K_{h}\\ \;+\;\alpha_{2}\gamma \ln\;K_{l}-\ln(1-\xi ) \end{array}$$

where *g*_*E*_, *h, N, K*_*h*_, *K*_*l*_, and ξ are the proportion of environmental change, the environmental protection technology level, natural resource exploitation, the credit of polluting enterprises, the credit of cleaning enterprises, and the credit of polluting enterprises. A positive increase of *g*_*E*_ means that the environment is better. Equation (8) shows that the input of credit resources into green enterprises *l* can increase their output, while decreasing resource consumption and environmental pollution.

## 4. Method

### 4.1. Quantile regression model

This paper constructs a benchmark regression model of green credit on social health costs, as follows:


(9) $$\begin{array}{lll} Health_{it} & = & \alpha_{0}+\alpha_{1}Credit_{it}+\alpha_{i}Control_{it}+\lambda_{i}+\varepsilon_{it}i=1,2,\cdots \;,N \end{array}$$

$$\begin{array}{lll} t & = & 1,2,\cdots \;,T \end{array}$$

where *Health*_*it*_ is the social health cost, *Credit*_*it*_ is green credit, *Control*_*it*_ represents the control variable, α_1_ is the effect of green credit on social health costs, λ_*i*_ represents the fixed effect, and ε_*it*_ represents the residual and follows a normal distribution.

When the benchmark regression Equation (9) is estimated, a traditional OLS can obtain only the impact of explanatory variables on the expected value of the explained variables. The OLS cannot analyze the influence of the distribution law. The QR method proposed by Koenker and Bassett (49) can solve this problem. The method assumes that the quantile of the conditional distribution of the dependent variable is a linear function of the independent variable, resulting in the construction of the QR of the dependent variable. Moreover, a QR can determine whether the independent variable has a specific regression curve on each characteristic quantile of the dependent variable. Therefore, compared with an OLS, a QR can more comprehensively describe the influence of the independent variables on the variation range of the dependent variables and show the conditional distribution shape. On the other hand, a QR uses the weighted average of the residual absolute value as the minimized objective function. Compared with an OLS, a QR is not easily affected by extreme values, and the estimation result is more robust.

Because the differences in economic development levels and policy implementation effects of regions in China, this paper uses a QR to analyze more comprehensively the impact of green credit on social health costs. The QR is defined as:


(10) $$\begin{array}{l} Quant_{\theta }\left(Health_{it}|X_{it}\right)=\alpha^{\theta }X_{it} \end{array}$$

where *X*_*it*_ includes *Credit*_*it*_ and *Control*_*it*_, α^θ^ is the coefficient variable, and *Quant*_θ_(*Health*_*it*_|*X*_*it*_) is the conditional quantile corresponding to quantile θ (0 < θ < 1)of the social health cost given X. Coefficient vector α^θ^ corresponding to θ is realized by minimizing the absolute deviation. The estimated value of the regression coefficient should minimize the following objective functions:


(11) $$\begin{array}{l} \alpha^{\theta }=\mathrm{argmin}\{\sum_{it,Health_{it}\geq X_{it}\alpha }\theta |Health_{it}-X_{it}\alpha \\ \;+\sum_{it,Health_{it}\geq X_{it}\alpha }\left(1-\theta \right)|Health_{it}-X_{it}\alpha \} \end{array}$$

Obviously, different regression lines are obtained for different θ. With a value of θ from 0 to 1, we obtain all the trajectories of the conditional distribution of explained variable *Health*_*it*_, that is, a cluster of curves. Therefore, compared with the OLS mean regression with only one regression curve, the QR can more fully reflect the relationship between the model variables.

### 4.2. Mediation effect model

Assuming that the variables are continuous and standardized, the regression model considering environmental pollution (*Env*) as a mediating variable is as follows:


(12) $$\begin{array}{lll} Env_{it} & = & \gamma_{0}+\gamma_{1}Credit_{it}+\gamma_{i}Control_{it}+\lambda_{i}+\nu_{it} \end{array}$$

$$\begin{array}{lll} Health_{it} & = & \beta_{0}+\beta_{1}Credit_{it}+\beta_{2}Env_{it}+\beta_{i}Control_{it}+\lambda_{i} \end{array}$$

(13) $$\begin{array}{ll} + & \mu_{it} \end{array}$$

In Equation (12), γ_1_ is the effect of green credit on environmental pollution. In Equation (13), β_2_ is the effect of mediating variable environmental pollution on social health costs after controlling for green credit. λ_*i*_ represents the fixed effect, and μ_*it*_ *and v*_*it*_ represent the residuals, assuming that they all follow a normal distribution and are independent of one another. Substituting Equation (13) into Equation (12), we obtain:


$$\begin{array}{lll} Health_{it} & = & k_{0}+\left(\beta_{1}+\gamma_{1}\cdot \beta_{2}\right)Credit_{it}+k_{i}Control_{it} \end{array}$$

(14) $$\begin{array}{ll} + & \nu_{it}\cdot \beta_{2}+\mu_{it} \end{array}$$

where γ_1_·β_2_ is the mediating effect of green credit on social health costs, β_1_ is the direct effect of green credit on social health costs, and β_1_+γ_1_·β_2_ is the total effect of green credit on social health costs. It can be concluded that α_1_ = β_1_+γ_1_·β_2_. Therefore, the mediating effect of environmental pollution can be estimated to observe its role in the social health costs of green credit.

## 5. Data

This paper is based on the data of 30 administrative regions of mainland Chinese provinces for 2005–2020. Tibet was excluded because of a lack of data. The data sources are the China Statistical Yearbook, China Environmental Statistical Yearbook, China Urban Statistical Yearbook, and Economic Census Yearbook. In 2005, BankTrack investigated the implementation of the Equator Principles^3^ and thought that some institutional members were just “green washing.” This phrase means that some enterprises do not consider environmental factors in their daily business activities and investment decisions but still aim to maximize their profits and is a phenomenon that has aroused worldwide attention. In 2007, China issued the Opinions on Implementing Environmental Protection Policies and Regulations to Prevent Credit Risk, which noted that green credit, as an economic means, has become a primary form of pollution reduction. In 2012, the China Banking Regulatory Commission (CBRC) issued Guidelines on Green Credit, which put forward clear requirements for banking financial institutions to engage in green credit and to vigorously promote energy conservation, emissions reductions, and environmental protection. In 2022, according to the CBRC, the balance of green credit at the end of the third quarter increased by 29.5% compared with the beginning of the year. With the orderly promotion of the banking industry, green credit developed rapidly. At present, there are three main ways to measure the green credit scale: the dummy variables of green credit policies (28), bank loans aimed at energy conservation and environmental protection (3), and the credit share of energy-intensive and highly polluting industries. As the dummy variables are more suitable for comparing green credit policies, and this paper uses provincial level data, the credit share can indirectly and partially reflect the regional green credit development level (17, 50). The credit share refers to the ratio of the interest expenditures of the six high energy–consuming industries^4^ to the industrial output. We used the negative value of this ratio to measure green credit. Green credit can affect financial funds or residents' health in a variety of ways, while the deterioration of the environment threatens residents' health, so more medical financial expenditures are needed. We chose per capita financial expenditures on medical care to measure social health costs. Through a theoretical analysis, environmental pollution, as a mediating variable, has been found to play a substantial role in the model of green credit affecting health. Based on the industrial wastewater, industrial SO_2_, and industrial dust discharges, we used the entropy method to calculate the comprehensive environmental index.

Among the control variables, we selected three presentation modes to govern the environment: the treatment number of industrial waste gas (Gas) and water facilities (Water) and the comprehensive utilization amount of industrial solid waste (Solid). The financial expenditure on environmental governance (Fee) represents various levels of regional support for environmental protection. The health status is affected by population aging. Generally, places with a large number of older adult people (Old) require more medical and health expenditures, so we chose the older adult dependency ratio to measure the aging degree. In addition, the economic development level is an important factor affecting health expenditures and environmental pollution. We chose GDP per capita and took 2005 as the base period for the decrease.

The variables' descriptive statistics are shown in Table 1. The average value of social health costs were 24,900 yuan/person, with a large difference between the extreme values (8.622). The average level of green credit in various regions was 0.512, and the environmental pollution level was 0.539, with little difference between the extreme values. However, except for the variables old and GDP, the extreme values of the control variables differed greatly, a fact that can also be seen from their standard deviations.

**Table 1:** Variable descriptive statistics.

	**Variable**	**Unit**	**Numbers**	**Mean**	**Std**.	**Min**.	**Max**.
Dependent variable	Health	10,000 yuan/person	480	0.249	0.849	0.002	8.622
Independent variable	Credit	ratio	480	0.512	0.203	0.010	2.411
Mediating variable	Env	–	480	0.539	0.530	0.001	2.585
Control variable	Gas	set	480	8508.719	7932.646	332.000	57278.000
	Water	set	480	2586.198	2250.746	103.000	10608.000
	Solid	10,000 tons	480	5794.541	4778.710	87.000	25230.000
	Fee	10,000 yuan/person	480	375.481	1716.833	0.551	37432.540
	Old	ratio	480	13.778	3.360	7.440	25.480
	GDP	10,000 yuan/person	480	4.421	2.785	0.531	16.493

**Figure 1:**
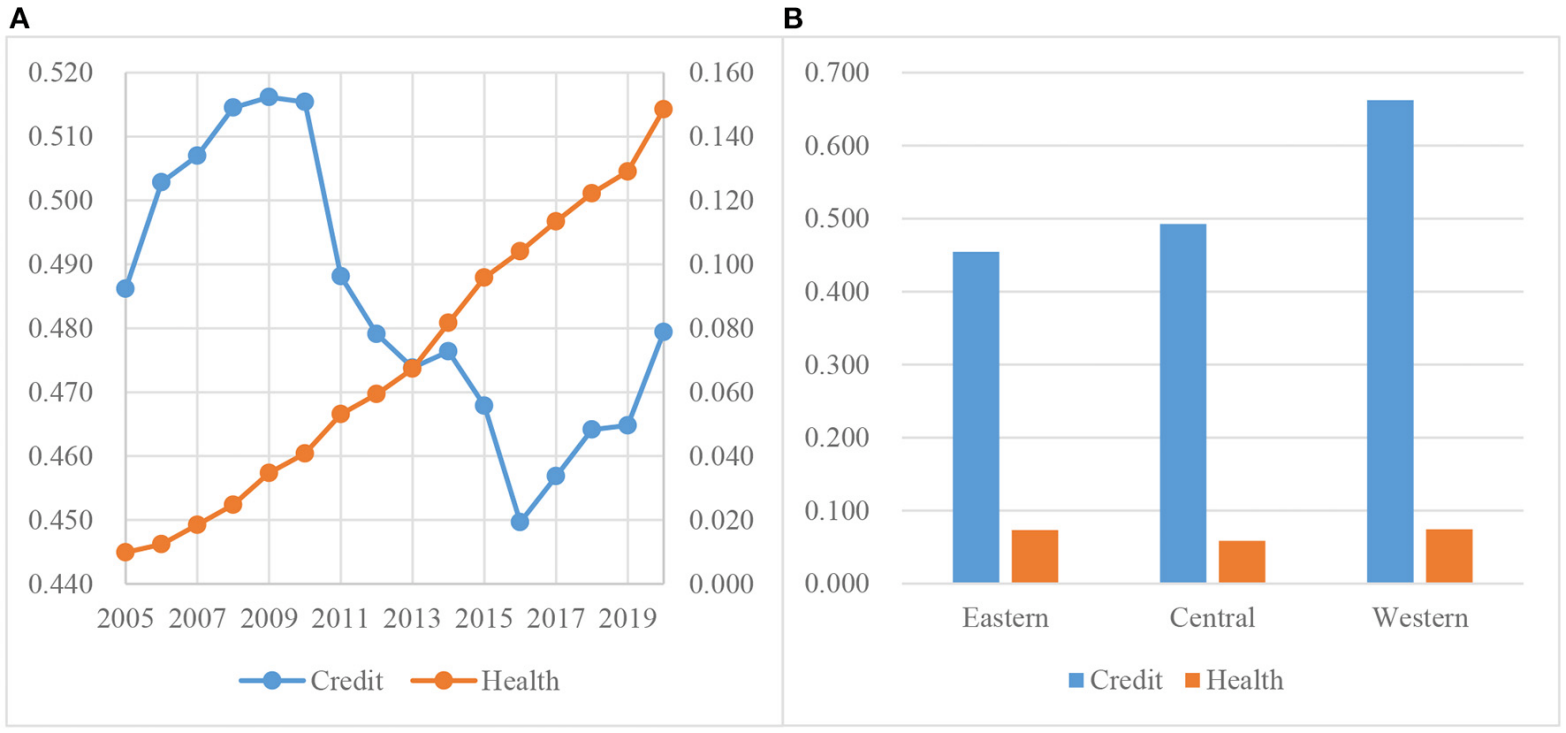
**(A, B)** Trend and regional difference in credit and health.

Figure 1A shows the trend in national green credit and social health costs from 2005 to 2020. The two variables developed in different directions. Social health costs increased over time, from 0.01 in 2005 to 0.148 in 2020^5^, an increase of 14%, with an average annual increase of 0.875%. This result shows that China's financial investment in social health expenditures has increased, especially since COVID-19 in 2019, and the trend is obviously increasing, which introduces great challenges to finance. Green credit fluctuated greatly during the review period. It increased from 2005–2009 and declined significantly from 2009–2013, from 0.516 to 0.474, for a decline of 8.94%. It increased slightly in 2013–2014 and then began to decrease sharply, rebounding until 2016 and reaching 0.479 in 2020. However, this figure was still far below the peak of 0.516, reached in 2009.

To observe the green credit and social health costs in different regions, according to the economic geographical location, the whole sample is divided into the Eastern, Central, and Western regions^6^. Observing the regional differences in green credit in Figure 1B, we found that the financial institutions in the Eastern region released the least amount of green credit, followed by the Central region and the Western region. This result may be due to the continual migration of polluting enterprises from the Eastern region to inland areas. Most of such enterprises are high-tech industries such as tertiary ones. Regarding the regional differences in social health costs, we found that the Central region had the least financial expenditures on health care, while the difference between the Eastern and Western regions was practically negligible. This result shows that the fiscal expenditures on health in the Central region lag behind those in the Eastern and Western regions, but the difference is not significant.

## 6. Empirical analysis

### 6.1. Quantile regression estimation results

To determine the function form, the Hausman test result for Equation (9) was 26.55, and the *p*-value was 0.00. Therefore, it is appropriate to reject the original assumption that individuals are random effects and to select a fixed effect model. For comparison with the panel QR results, we also show a fixed effect model estimation (Column 7). In the panel quantile estimation (Table 2), the five representative quantiles of 10, 25, 50, 75, and 90% are selected.

**Table 2:** Quantile regression results of green credit on social health costs.

	**10%**	**25%**	**50%**	**75%**	**90%**	**FE**
Credit	−0.162 [−0.9]	−0.240^*^ [−1.78]	−0.338^***^ [−2.97]	−0.443^***^ [−2.93]	−0.641^**^ [−2.19]	−0.347^***^ [−3.23]
Gas	−0.811^***^ [−3.32]	−0.615^***^ [−3.43]	−0.365^***^ [−2.37]	−0.100 [−0.49]	0.399 [1.02]	−0.344^***^ [−2.66]
Water	−0.004 [−0.02]	−0.146 [−1.08]	−0.327^***^ [−2.80]	−0.519^***^ [−3.39]	−0.881^***^ [−2.96]	−0.343^***^ [−3.39]
Solid	0.147 [1.15]	0.034 [0.37]	−0.108 [−1.34]	−0.260^***^ [−2.47]	−0.546^***^ [−2.66]	−0.121^*^ [−1.93]
Fee	−0.170 [−1.16]	−0.104 [−0.95]	−0.019 [−0.21]	0.071 [0.57]	0.239 [1.01]	−0.012 [−0.16]
GDP	0.770^***^ [3.99]	0.789^***^ [5.45]	0.812^***^ [6.66]	0.837^***^ [5.15]	0.884^***^ [2.82]	0.814^***^ [8.39]
Old	−0.674^**^ [−1.89]	−0.539^**^ [−2.02]	−0.367 [−1.63]	−0.184 [−0.62]	0.159 [0.28]	−0.352^*^ [−1.72]
Intercept	–	–	–	–	–	3.471^***^ [4.35]

*, **, *** represent 10, 5, 1% significance levels, respectively.

The elasticity coefficient of green credit was negative in both the OLS regression and each quantile level, all of which except the 10% quantile level passed the 10% significance level test. Since 2005, the green credit of various institutions in China decreased social health costs and relieved financing pressures on health expenditures. The change trend of each quantile reveals that as the quantile increased, the absolute value of the green credit coefficient gradually increased from 0.162 to 0.641. This change shows that for provinces with high per capita financial health expenditures, green credit plays a greater role in decreasing social costs (51, 52). Green credit plays an indispensable role in decreasing financial expenditures and protecting public health. For example, the financial industry quickly took measures to adjust credit to prevent and control COVID-19 and supported various industries as they attempted to resume production. The main methods include deferred repayment, the reduction of late fees, the reduction of interest rates for small and micro enterprises, and the establishment of special funds to match targets. In early 2020, China Development Bank launched the CDB Anti -epidemic Special Bond to effectively provide financial support to fight the epidemic. All the above measures can decrease social health expenditures. The literature also supports the conclusion of this paper by affirming the important contribution of green credit in decreasing public health expenditures (10, 11). In addition, Hu et al. (53) used a QR to show that the increasing effect of green credit on green total factor productivity increased as the latter increased.

The QR results of each control variable show that the first 50% quantile, the elasticity coefficient of industrial waste gas was significantly negative. Industrial wastewater was negative in all fractions, but only the last 50% of the quantiles passed the 10% significance level test, and industrial solid waste was significantly negative in the last 75% quantile. Although the significance of industrial waste differs, in general, the treatment of industrial wastewater, waste gas and solid waste can have a positive effect on decreasing social health costs. The elasticity coefficient of fiscal environmental expenditures in the first 25% quantile was negative but not significant. The elasticity coefficient of GDP was positive in all quantiles, passing the 10% significance level. This result indicates that regions with high economic development levels and high also have relatively high per capita medical and health expenditures. As the quantile level increased, the elasticity coefficient increased from 0.770 to 0.884. This result means that economic growth has a greater role in decreasing social health costs in regions where such costs are high. The elasticity coefficient of population aging was significantly negative in the first 50% quantile. This result indicates that as population aging intensifies, per capita medical health expenditures did not increase. The impact is limited of aging on the increase in medical and health expenditures, especially in rural areas. Because economic development is at a low level, the demand for medical services for the older adult population has not been effectively met due to the imperfect medical security system and the lack of medical resources. Therefore, at this stage, rural aging has not affected medical expenditures, and even as the aging degree improves, self-funded medical health expenditures have decreased.

In the fixed effect regression model, the regression coefficient of green credit was −0.347, which was significantly negative at the 1% level, indicating that implementing green credit can decrease social health costs. The elasticity coefficient of each control variable was also numerically equivalent to the mean QR value, which verifies the robustness of the results.

### 6.2. Regional heterogeneity analysis

This paper divides the whole sample into three regions—Eastern, Central, and Western—and comparatively analyzes the regional differences. Table 3 lists only the elasticity coefficients of green credit under different quantiles and fixed effects, absent the control variables.

**Table 3:** Regional heterogeneity.

	**Eastern**	**Central**	**Western**
10%	−0.557^***^ [−2.77]	−0.270 [−1.19]	0.337 [0.71]
25%	−0.621^***^ [−4.24]	−0.295^*^ [−1.79]	0.209 [0.73]
50%	−0.698^***^ [−5.85]	−0.322^**^ [−2.33]	0.107 [0.58]
75%	−0.797^***^ [−4.62]	−0.363^*^ [−1.70]	0.024 [0.11]
90%	−0.854^***^ [−3.76]	−0.379^*^ [−1.83]	−0.032 [−0.12]
FE	−0.702^***^ [−5.42]	−0.326^**^ [−2.07]	0.125 [0.62]
Samples number	176	128	176

*, **, *** represent 10, 5, 1% significance levels, respectively.

Table 3 shows that the fixed effect coefficient was basically maintained at the average level of each quantile and thus verifies the robustness of the QR results. The elasticity coefficient of green credit in the Eastern region was negative in all quantiles. As the quantile increased, the absolute value of the elasticity coefficient increased from 0.557 to 0.854, all of which passed the 1% significance level test. This result verifies that the increase of green credit can decrease social health costs, and the effect is greater and more significant than is that under the full sample condition. The elasticity coefficients of green credit in the Central region were all significantly negative (except for the low quantile of 10%). Furthermore, as the quantile increased, the absolute value increased from 0.270 to 0.379. This result verifies the negative relationship between green credit and social health costs, but the negative effect is lower than was that in the Eastern region. Except for the 90% high quantile in the Western region, the elasticity coefficients of the quantiles were positive and not significant, indicating that no effect was found for green credit decreasing social health costs in the Western region. In short, the impact of green credit on social health costs shows an obvious geographical ladder distribution, with the largest effect in the Eastern region, followed by the Central region, and an as-yet-to-be-found effect in the Western region. This conclusion is consistent with Zhong's (31) view that the development of digital finance in the Eastern region is generally ahead of other regions, and the environmental improvement is more effective. Li et al. (7) also believed that green credit improved the total factor productivity only in the Eastern region but had little impact in other regions, so the promotional effect of financial and legal developed areas would be more effective. Therefore, the Chinese government should encourage the implementation of regionally differentiated green credit policies, increase investment in less-developed regions, and pay attention to the efficiency of capital use.

### 6.3. Verification of the mediating effect of the environment

In the process of decreasing social health costs, green credit inevitably considers the important role played by environmental pollution. Most researchers recommend using the bootstrap method for a mediating effect analysis (54, 55) as it corrects the interval estimation error by adjusting the percentile of the sequence interval. Regarding H0, γ_1_·β_2_ = 0, when the sample size is 5,000, the results of the non-parametric percentile bootstrap method are shown in Table 4. Under the 95% confidence interval, the intermediate test results did not include 0 (LLCI = −1.045, ULCI = −0.683), so H0 is rejected, indicating that the mediating effect of environmental pollution was significant, and the size of the mediating effect was −0.864. In addition, after controlling for the mediating variable environmental pollution, green credit did not have a significant impact on social health costs, and the confidence interval included 0 (LLCI −0.012, ULCI = 0.053). Therefore, environmental pollution plays a mediating role in the pathway of green credit to social health cost and is the only mediating variable.

**Table 4:** Bootstrap test results.

	**Coef**.	**BootSE**	**LLCI**	**ULCI**
Direct effect	0.011	0.012	−0.012	0.053
Indirect effect	−0.864^***^	0.092	−1.045	−0.683

*, **, *** represent 10, 5, 1% significance levels, respectively; LLCI and ULCI are the lowest and highest values of the confidence interval; BootSE is the standard error.

The estimated results for Equations (12) and (13) are shown in Table 5, which examines the mediating effect of environmental pollution. Equation (12) takes environmental pollution as the explained variable. The elasticity coefficient of green credit was −0.269, passing the 1% significance level test, indicating that green credit plays an important role in environmental protection. On the one hand, through green credit tools, the banking industry has increased support for carbon emissions trading and environmental liability insurance, which have alleviated human damage to the environment. On the other hand, green credit can effectively encourage enterprises to carry out green technology innovation. For example, enterprises widely use clean energy, clean materials, and green technologies and processes to replace their original ones, ultimately decreasing the risk of environmental pollution. Therefore, green credit is a financial activity that supports environmental improvement, responds to climate change, and effectively uses resources and is an important guarantee for sustainable economic development.

**Table 5:** Regression results of the mediating effect.

	**Dependent variable: Env**	**Dependent variable: Health**
Credit	−0.969^***^ [−3.40]	−0.298^**^ [2.14]
Env	–	0.183^***^ [2.86]
Intercept	1.405 [1.37]	5.747^**^ [1.99]
Control	Yes	Yes
*F*	18.41	17.37
Adj-*R*2	0.572	0.684

*, **, *** represent 10, 5, 1% significance levels, respectively.

The setting of Equation (13) in Table 5 takes social health cost as the explained variable. The elasticity coefficient of the green credit was −0.298, while environmental pollution was 0.183, which were all significant at the 1% level. This result not only supports the role of green credit in decreasing health costs but also shows that the increase of industrial waste emissions might increase the social expenditures on health. We analyze the conclusion from both psychological and physical aspects. On the one hand, environmental pollution has a negative effect on public mental health, decreasing subjective wellbeing and mental health (56). On the other hand, environmental pollution accelerates the depreciation of physical health, decreases the productivity of personal exercise used to increase health investments, and negatively affects the public's physical health. Guo et al. (57) proved that industrial structure upgrades and environmental investment play positive intermediary roles between green credit and green economy efficiency. Moreover, Zeng et al. (17) and Muhammad et al. (18) supported the research results using different national data sources and methods.

In summary, green credit has a direct effect on social health costs if −0.298, an indirect effect of −0.049 (=-0.269^*^0.183), and a total effect for −0.347 [=-0.298+(−0.269^*^0.183)]. The calculated total effect is consistent with the elasticity coefficient of green credit in the overall regression, Equation (9) in Table 2 (Column 7). This result shows that green credit directly impacts social health costs and indirectly affects residents' health through environmental pollution.

### 6.4. Robustness check

Table 2 lists the fixed effect regression results. The sign of the green credit in the fixed effect model was consistent with the QR, and the size was basically the same as it was in the average quantile, which indicates the robustness of the QR model. In this section, we measure the explained variable social health cost by per capita medical care expenditures to further test the robustness of this research object.

In Table 6, after the explained variable was replaced, the coefficient of green credit did not change much compared with the result in Table 2. Only the size or significance slightly decreased or improved, a fact that does not affect the conclusion of this paper. The elasticity coefficient of green credit was negative at the 10% significant level, which further indicates that green credit can decrease social health expenditures.

**Table 6:** Quantile regression results of green credit on health care expenditures.

	**10%**	**25%**	**50%**	**75%**	**90%**	**FE**
Credit	−0.236^***^ [−3.86]	−0.574^***^ [−6.92]	−1.019^***^ [−11.5]	−1.463^***^ [−10.71]	−1.854^***^ [−8.52]	−1.006^***^ [−15.61]
Gas	−0.565^***^ [−2.66]	−0.464^***^ [−3.06]	−0.332^***^ [−2.92]	−0.200 [−1.30]	−0.083 [−0.37]	−0.352^***^ [−3.33]
Water	−0.508^***^ [−2.73]	−0.573^***^ [−4.31]	−0.658^***^ [−6.61]	−0.743^***^ [−5.51]	−0.817^***^ [−4.14]	−0.644^***^ [−7.63]
Solid	0.291^***^ [2.70]	0.275^***^ [3.56]	0.254^***^ [4.42]	0.233^***^ [2.98]	0.215^*^ [1.87]	0.270^***^ [4.75]
Fee	−0.075 [−0.59]	−0.072 [−0.78]	−0.067 [−0.98]	−0.062 [−0.67]	−0.058 [−0.42]	−0.029 [−0.49]
GDP	−0.050 [−0.22]	−0.149 [−0.91]	−0.278^**^ [−2.27]	−0.407^**^ [−2.46]	−0.521^**^ [−2.15]	−0.248^**^ [−2.32]
Old	−0.587 [−1.57]	−0.682^**^ [−2.54]	−0.807^***^ [−4.03]	−0.931^***^ [−3.42]	−1.040^***^ [−2.61]	−0.773^***^ [−4.59]
Intercept	–	–	–	–	–	10.338^***^ [11.97]

*, **, *** represent 10%, 5%, 1% significance levels, respectively.

## 7. Conclusion

Green credit is an innovative financial concept and reflects the sustainable development of an economy and a society. Based on data from 30 provinces in China from 2005 to 2020, this paper examines the different impacts of green credit on social health costs using a QR. In addition, we examine the mediating role of environmental pollution in the impact path and mainly draw the following conclusions. The implementation of green credit policies can decrease social health costs. As the quantile improves, the absolute value of the green credit coefficient gradually increases. This change shows that as per capita financial health expenditures increase, green credit plays a greater role in decreasing social costs. This effect is strongest in the Eastern region and may be related to the economic development level. In addition, this paper confirms the mediating effect of environmental pollution and the fact that it is the only mediating variable, which has also been proven by the green credit and health general equilibrium model. Therefore, green credit can relieve financial pressure through social financing, release more funds for public health expenditures, and affect public health by improving the environmental quality.

The government should make full use of financial supports to help build a human health community and should improve the emergency management capabilities for major public health events as soon as possible. Policy-makers need to fully support internet green finance led by green credit, encourage the diversified development of green credit products, and increase the promotion of green credit products. At the same time, banking institutions should strengthen the positive innovation of green credit products, expand the modes of public participation, respond to the rapidly changing market, and decrease government pressure. In addition, green finance is the embodiment of the green development concept and can protect the human living environment and decrease the impact of pollution on residents' health (58). Therefore, financial institutions can develop personalized green credit products for specific pollutant types or industries. These practices will help to improve financial institutions' efficiency as they undertake environmental responsibilities and decrease costs.

## 8. Limitations

The impact of green finance on residents' health is not limited to green credit. Because green credit accounts for a large proportion of green finance, the scope of this paper is green credit. As green stocks, bonds, trusts, etc. gradually develop green finance's research objects should be gradually expanded to more comprehensively analyze green finance's impact on residents' health.

## Data availability statement

The original contributions presented in the study are included in the article/Supplementary material, further inquiries can be directed to the corresponding author.

## Author contributions

YR: conceptualization, methodology, software, data curation, and writing—original draft preparation. JH: visualization and investigation and writing—reviewing and editing. Both authors contributed to the article and approved the submitted version.

## Footnotes

^1^Data source: The Lancet.

^2^See Appendix A for details.

^3^According to the policy guidelines of the International Finance Corporation and the World Bank, the financial industry has established benchmarks aimed at judging, assessing, and managing environmental or social risks in project financing to increase the social responsibility of the banking industry and improve the increasingly stressed environment.

^4^The six high-energy consuming industries include petroleum processing, coking and nuclear fuel processing, chemical raw materials and chemical products manufacturing, non-metallic mineral products, ferrous metal smelting and rolling processing, nonferrous metal smelting and rolling processing, and the production and supply of electricity and heat.

^5^The health data is based on the secondary axis.

^6^Eastern region: Liaoning, Beijing, Tianjin, Shanghai, Hebei, Shandong, Jiangsu, Zhejiang, Fujian, Guangdong, Guangxi, and Hainan; Central region: Heilongjiang, Jilin, Shanxi, Inner Mongolia, Anhui, Henan, Huzibei, Hunan, and Jiangxi; and Western region: Chongqing, Sichuan, Yunnan, Guizhou, Tibet, Shaanxi, Gansu, Qinghai, and Ningxia.
