# Supplementary material for: How can green credit decrease social health costs? The mediating effect of the environment
Source: Front Public Health. 2023 Jan 20;11:1121154. doi: 10.3389/fpubh.2023.1121154 (PMC9895397; doi:10.3389/fpubh.2023.1121154)
Supplement: Supplementary file 1 [file Data_Sheet_1.pdf]

Appendix A

We expand the financial sector based on neoclassical growth theory with resource constraints. Suppose that an economy has two enterprise types that provide intermediate products. *h* represents the polluting enterprises, which is characterized by heavy assets, such as steel and petrochemical; *l* represents the clean enterprises, which are characterized by light assets and high technology, such as electronic components. The production functions of the two types of enterprises are:

$$Y_h = N^{\beta_1} K_h^{\beta_2} \ ; Y_l = A(t) K_l^{\gamma} \tag{A-1}$$

where *h* enterprises consume natural resources *N* and material capital K_h, *l* enterprises do not consume natural resources (actually, they consume fewer, a fact that is simplified here), and use only healthy human capital H and material capital K_l, with technological progress parameter A. In the consideration of technological progress, A is a function of time *t*. Production is also affected by the environment. Following the assumption of Bowenberg and Simus (1995), P represents pollution, and as it deepens, the output decreases more. Thus, the production function is as follows:

$$Y = Y_h^{\alpha_1} Y_l^{\alpha_2} P^{-\alpha_3}, \alpha_1, \alpha_2, \alpha_3 > 0 \tag{A-2}$$

Environment E can be damaged by production activities and decline with pollution. Therefore, the dynamic equation of the environment can be written as:

$$\dot{E} = \theta E - \delta P \tag{A-3}$$

$$P = \frac{N}{h} \tag{A-4}$$

where θ and δ are influence coefficients. *h* refers to environmental protection technical parameters. Under the same consumption, enterprises with more advanced environmental protection technologies produce less pollution.

People prefer both consumption and a good environment. We continue to use the additive fixed elastic utility function, and the instantaneous utility function is:

$$U = \frac{C^{1-\sigma}-1}{1-\sigma} + \frac{E^{1+\omega}-1}{1+\omega} \tag{A-5}$$

where C is consumption, σ is the relative risk aversion coefficient and the reciprocal of intertemporal substitution elasticity, ω is related to people's preference for the environment, and an increase in ω indicates that people pay more attention to the environment. Under these model assumptions, social planners face the following optimization problems:

$$max \int_0^{+\infty} \left(\frac{C^{1-\sigma}-1}{1-\sigma} + \frac{E^{1+\omega}-1}{1+\omega} \right) e^{-\rho t} dt \tag{A-6}$$

$$s.t.\begin{cases} Y = Y_h^{\alpha_1} Y_l^{\alpha_2} P^{-\alpha_3} = BN^{\alpha_1\beta_1-\alpha_3} K^{\alpha_1\beta_2+\alpha_2\gamma} \\ P = N/h \\ \dot{E} = \theta E - \delta P \\ \dot{K} = Y - C \end{cases} \tag{A-7}$$

where ρ is the first-order correlation coefficient of the random error term. Define control variables C and N and state variables E and K to solve the optimal control problem, and we obtain the first order condition:

$$\lambda_1 = C^{-\sigma} \tag{A-8}$$

$$\lambda_1 \mu_1 \left(\frac{Y}{N}\right) = \lambda_2 \left(\frac{\delta}{h}\right) \tag{A-9}$$

The Euler equation is:

$$\lambda_1 = \rho\lambda_1 - \lambda_1\mu_2 \left(\frac{Y}{K}\right) \tag{A-10}$$

$$\lambda_2 = \rho\lambda_2 - E^{\omega} - \theta\lambda_2 \tag{A-11}$$

In the steady state, there is $g_E = g_N$, $g_Y = g_C = g_K$. g indicates the variable change, and if it increases in the positive direction, the environment improves. We take the logarithm of both sides of the first-order condition and the Euler equation, and take the derivative of time to obtain:

$$g_{\lambda_1} = -\sigma g_C \tag{A-12}$$

$$g_{\lambda_1} = \rho - \mu_2 \left(\frac{Y}{K}\right) \tag{A-13}$$

In the steady state, we obtain:

$$g_E = \frac{(\sigma-1)}{(1+\omega)} \frac{1}{\sigma} \left[\mu_2 \left(\frac{Y}{K}\right) - \rho\right] \tag{A-14}$$

Green credit ξ acts on the material capital of environmental protection enterprises, mean $K_l = \xi K$. Substituting the production Equation (A-2) considering green credit into (A-14) while taking logarithms, we obtain:

$$g_E = \frac{(\sigma-1)}{(1+\omega)} \frac{1}{\sigma} \left[\mu_2 h^{\alpha_3} A^{\alpha_2} N^{\mu_1} K_h^{\mu_2-1-\alpha_2\gamma} K_l^{\alpha_2\gamma} (1-\xi) - \rho\right] \tag{A-15}$$

$$ln\, g_E = \phi + \alpha_3\, ln\, h + (\alpha_1\beta_1 - \alpha_3)\, ln\, N + (\alpha_1\beta_2 - 1)\, ln\, K_h + \alpha_2\gamma\, ln\, K_l - ln(1-\xi) \tag{A-16}$$

where g_E, h, N, K_h, K_l, and ξ are the proportion of environmental change, the environmental protection technology level, natural resource exploitation, the credit of polluting enterprises, the credit of cleaning enterprises, and the credit of polluting enterprises. The positive increase of g_E means that the environment is better.
